# QuaDMutNetEx: a method for detecting cancer driver genes with low mutation frequency

**DOI:** 10.1186/s12859-020-3449-2

**Published:** 2020-03-23

**Authors:** Yahya Bokhari, Areej Alhareeri, Tomasz Arodz

**Affiliations:** 1 Department of Computer Science, College of Engineering, Virginia Commonwealth University, 401 W. Main St., Richmond, VA 23284 USA; 2 Department of Biostatistics and Bioinformatics, King Abdullah International Medical Research Center, Riyadh, Saudi Arabia; 3 King Saud bin Abdulaziz University for Health Sciences, Riyadh, Saudi Arabia; 4 College of Applied Medical Sciences, King Saud bin Abdulaziz University for Health Sciences, Riyadh, Saudi Arabia; 5 King Abdullah International Medical Research Center, Riyadh, Saudi Arabia

**Keywords:** Somatic mutations, Cancer pathways, Driver mutations, Protein-protein interaction networks

## Abstract

**Background:**

Cancer is caused by genetic mutations, but not all somatic mutations in human DNA drive the emergence or growth of cancers. While many frequently-mutated cancer driver genes have already been identified and are being utilized for diagnostic, prognostic, or therapeutic purposes, identifying driver genes that harbor mutations occurring with low frequency in human cancers is an ongoing endeavor. Typically, mutations that do not confer growth advantage to tumors – passenger mutations – dominate the mutation landscape of tumor cell genome, making identification of low-frequency driver mutations a challenge. The leading approach for discovering new putative driver genes involves analyzing patterns of mutations in large cohorts of patients and using statistical methods to discriminate driver from passenger mutations.

**Results:**

We propose a novel cancer driver gene detection method, QuaDMutNetEx. QuaDMutNetEx discovers cancer drivers with low mutation frequency by giving preference to genes encoding proteins that are connected in human protein-protein interaction networks, and that at the same time show low deviation from the mutual exclusivity pattern that characterizes driver mutations occurring in the same pathway or functional gene group across a cohort of cancer samples.

**Conclusions:**

Evaluation of QuaDMutNetEx on four different tumor sample datasets show that the proposed method finds biologically-connected sets of low-frequency driver genes, including many genes that are not found if the network connectivity information is not considered. Improved quality and interpretability of the discovered putative driver gene sets compared to existing methods shows that QuaDMutNetEx is a valuable new tool for detecting driver genes. QuaDMutNetEx is available for download from https://github.com/bokhariy/QuaDMutNetExunder the GNU GPLv3 license.

## Background

Cancer driver mutations are DNA changes that are causally implicated in oncogenesis [1, 2]. Typically between two and eight mutations, targeting several cellular pathways, are needed for cancer to develop [3]. To disrupt a single pathway or a group of functionally related genes in a way that promotes cancer growth, often only one mutation is needed [4–6].

Most DNA mutations are not cancer drivers. Mutations in DNA accumulate throughout life – for example, comparing skin or gastrointestinal epithelium cells in cancer samples from patients 85 and 25 years old showed that the younger patient on average has half the number of mutation compared to the older patient. More than half of all mutations found in cancer tissue are estimated to have occurred before the start of the disease [7]. Additionally, mutation rate tends to increase in cancer cells [8], although it can differ significantly even among subclones within the tumor [9]. Most of these new random mutations do not contribute to the progression of the disease. An analysis of large number of cancer samples gathered in the Cancer Genome Atlas (TCGA) [10] shows that the total number of mutations present in a tumor tissue from a single patient can range from 10 to more than 100, and only about 2 to 6 among them are driver mutations [11]. Hence, the majority of mutations present in a cancer tissue sample are passenger mutations, with no positive impact on oncogenesis. Due to the potential of using driver genes, that is, genes that harbor driver mutations, for therapeutic, prognostic, or diagnostic purposes, assembling a comprehensive catalogue of driver genes an important ongoing endeavor [12–14]. The main challenge in this task is discovering new driver genes while avoiding false positives stemming from the abundance of passenger mutations.

Statistical and computational methods for detecting driver genes often rely on finding certain pattern of mutations in a group of driver genes across a cohort of patients. To develop cancer, multiple cellular functions must be perturbed, and in different patients, different genes with the same function may be mutated. Often, the cancer develops and is detected before a second mutation in genes with a given function occurs. Thus, for a given cancer type, for a group of patients, each patient would have at least one mutation in a functionally-related group of driver genes, but rarely would have more than one mutation – the gene set exhibits mutual exclusivity pattern of mutations. Several methods detect a set of driver genes by quantifying mutual exclusivity, including Dendrix [15] and Multi-Dendrix [16], RME [17], CoMEt [18], TiMEx [19], MEMo [20], and our own method, QuaDMutEx [21]. An alternative approach involves knowledge of networks linking genes. Frequently mutated genes and their less-frequently mutated neighbors in known human gene- or protein-level pathways or networks are detected as drivers. Methods such as HotNet2 [22, 23], MEMo [20] and DriverNet [24] adopted the network-oriented driver detection approach.

Biological network connectivity and mutual exclusivity are both important sources of information in discovering driver genes. At the same time, both types of information must be used with caution. The available biological networks are incomplete and are expected to include false positives, which might affect the true structure of the network in a way that is unknown. Deviations from mutual exclusivity pattern are expected in individual patients, especially in slow-growing tumors where random mutations have more time to accumulate before cancer is detected. Therefore, an algorithm that uses biological networks and mutual exclusivity at the same time will be able to utilize two complementary, imperfect sources of information to improve the quality of the discovered putative driver gene sets.

We propose a tool, QuaDMutNetEx, which combines the network and exclusivity based approaches. As in our previous tool QuaDMutEx [21], the objective function that is used to select driver genes penalizes for any deviation from the mutual exclusivity pattern. Additionally, QuaDMutNetEx shows preference for genes that are connected in known human biological networks. Compared to mutual exclusivity-based tools such as QuaDMutEx or Dendrix, this additional source of information can help in finding rare driver mutations, for which neither the network connectivity and mutation frequency alone, nor exclusivity alone, are selective enough. The tool models both the network and the mutual exclusivity terms of the objective function as convex, quadratic terms, resulting in a binary quadratic problem, which is solved using our previously proposed technique of efficiently exploring the space of gene sets by using stochastic search through a series of globally optimal solutions to subproblems. Comparisons with existing state-of-the-art methods on four cancer datasets show that the approach of combining network and exclusivity approaches results in improved ability to detect highly connected, mutually exclusive rare driver genes.

## Results

We evaluated QuaDMutNetEx using its default parameters that have been selected experimentally: the maximum size of the gene set is *ν*=50; *k*=1, indicating equal preference for optimizing coverage and excess coverage; *C*=2.5; the network parameter was set to *α*=0.3; the number of iterations was set to *T*=10,000. In the network-connectivity term of the objective function, we used combined three human protein-protein interaction networks previously used in HotNet2 [23]. The first network is the iRefIndex network, which consists of 91,872 interactions among 12,338 proteins. The second network is MultiNet network which consists of 109,597 interactions among 14,445 proteins. The last network is HINT+HI2012 which is created by considering two interactome databases: HI-2012 data in human HI2 Interactome database, HI2012, and high-quality interactomes database, HINT. The HINT+HI2012 network consists of 40,783 interactions among 10,008 proteins.

We used four datasets to evaluate the proposed algorithm: triple-negative breast cancer (TN), glioblastoma multiforme (GBM), high-grade serous ovarian cancer (HGS), and another breast cancer (METABRIC) dataset (see Table 1). These datasets were previously used in evaluating the DriverNet tool. Following standard practice, known hypermutated genes such as mucins, titin, olfactory receptors, which are unlikely to play role in cancer, were removed [25].

**Table 1:** Summary of DriverDB tool datasets used in experimental validation of QuaDMutNetEx

Dataset	Samples (n)	Genes (p)	Mutations
TN: triple negative breast cancer	94	4594	6007
GBM: glioblastoma multiforme	120	3747	8141
HGS: high-grade serous ovarian cancer	316	13278	22897
METABRIC: breast cancer	696	13076	51255

### Quantitative and qualitative assessment of QuaDMutNetEx results

The results of the tests, presented in Table 2, show that the proposed method returns gene sets that are statistically significant at 0.05. To assess statistical significance of the results returned by QuaDMutNetEx, we used permutation test proposed in [15]. In short, we randomly permuted the patient-gene matrix in a way that preserves the number of mutations in each patient, and in each gene. This process results in a randomized dataset in which any correlations of mutations are only appearing by chance, but the gene mutation frequencies and patient mutation counts are the same as in the original dataset, which keeps the randomized dataset similar to the original. We created 256 randomized datasets and ran QuaDMutNetEx on each dataset. To obtain a *p*-value estimate, the final penalty score obtained from running QuaDMutNetEx on the original dataset was compared with the distribution of final penalty scores from running QuaDMutNetEx on the 256 randomized datasets.

**Table 2:** Quantitative characteristics of QuaDMutNetEx results

Dataset	Genes Found	Estimated *p*-value
TN	13	<0.004
GBM	6	<0.004
HGS	25	0.016
METABRIC	25	<0.004

Solutions for all four datasets are statistically significant at *p*<0.05

**Fig. 1:**
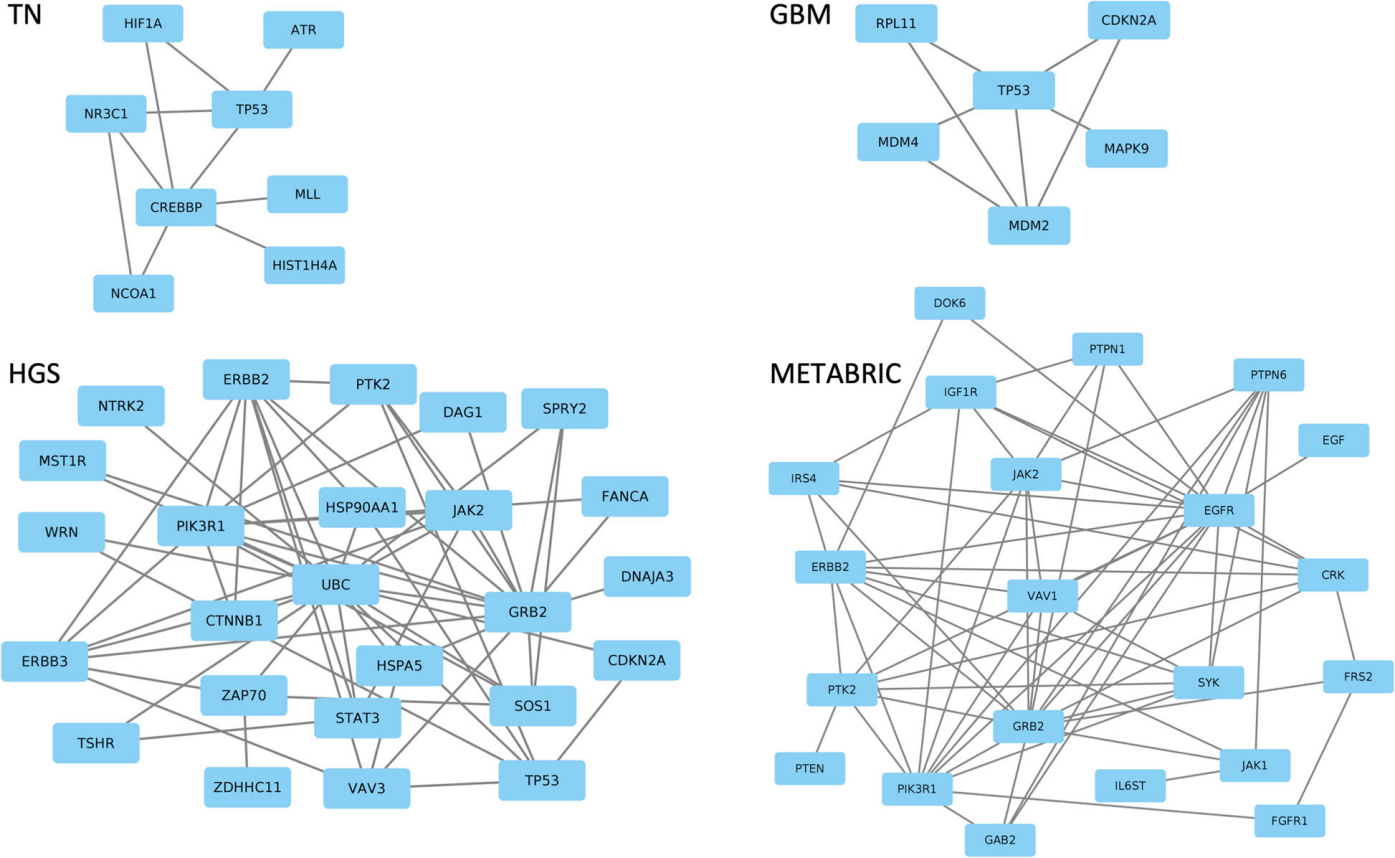
Known interactions between driver genes discovered by QuaDMutNetEx on the four datasets: TN: triple-negative breast cancer, GBM: glioblastoma multiforme, HGS: high-grade serous ovarian cancer, and METABRIC: breast cancer

The genes discovered by QuaDMutNetEx are presented in Table 3. To evaluate the gene’s driver status, we used COSMIC Cancer Gene Census database [26, 27] and the cancer driver gene database DriverDBv2 [28]. To check QuaDMutNetEx’s effectiveness in discovering rare cancer drivers, we focused on the genes in the solutions that are least frequently mutated in the datasets, and preformed literature review to analyze if these are true or false positives. Additionally, we visually evaluated the resulting gene networks – the largest connected component for each dataset is presented in Fig. 1. To show how inclusion of the network connectivity term in the objective function improves the solution, we have denoted genes found on the same datasets by our mutual-exclusivity-only method, QuaDMutEx. We see that most of the discovered driver genes, especially those mutated with low frequency, result from including the network connectivity term.

**Table 3:** Putative driver gene sets discovered by QuaDMutNetEx

Gene	c	D/R	QuanDMutEx	COSMIC	DDBv2	Gene	c	D/R	QuanDMutEx	COSMIC	DDBv2
**TN: Triple-negative breast cancer**											
TP53	35	R	✓	✓	✓	PARK2	6	∙	✓	∙	✓
ATR	4	D	∙	✓	✓	SAGE1	3	∙	✓	∙	✓
NR3C1	3	∙	✓	∙	✓	CREBBP	2	D/R	∙	✓	✓
DAPK1	2	∙	∙	∙	✓	NCOA1	2	D	∙	✓	✓
SLC39A7	2	∙	∙	∙	✓	IDH3B	2	∙	✓	∙	✓
HIST1H4A	2	∙	∙	∙	✓	HIF1A	2	D	∙	✓	✓
MLL	2	D	∙	✓	✓						
**GBM: Glioblastoma multiforme**											
CDKN2A	55	R	✓	✓	✓	TP53	38	R	✓	✓	✓
MDM2	13	D	∙	✓	✓	MDM4	5	D	∙	✓	✓
MAPK9	2	∙	∙	∙	✓	RPL11	2	∙	∙	∙	✓
**HGS: high-grade serous ovarian cancer**											
TP53	249	R	✓	✓	✓	SOS1	3	∙	∙	∙	✓
CTNNB1	2	D	∙	✓	✓	DAG1	2	∙	∙	∙	✓
ERBB2	2	D	∙	✓	✓	FANCA	2	R	∙	✓	✓
GRB2	2	∙	∙	∙	✓	PIK3R1	2	R	∙	✓	✓
TSHR	2	D	∙	✓	✓	DNAJA3	2	∙	∙	∙	✓
HSP90AA1	2	D	∙	✓	✓	HSPA5	2	∙	∙	∙	✓
MST1R	2	∙	∙	∙	✓	PTK2	2	∙	✓	∙	✓
STAT3	2	D	∙	✓	✓	UBC	2	∙	∙	∙	✓
VAV3	2	∙	∙	∙	✓	WRN	2	R	✓	✓	✓
ZAP70	2	∙	∙	∙	✓	ERBB3	2	D	∙	✓	✓
NTRK2	2	∙	∙	∙	✓	SPRY2	2	∙	∙	∙	✓
DHHC11	2	∙	∙	∙	✓	JAK2	2	D	∙	✓	✓
CDKN2A	2	R	∙	✓	✓						
**METABRIC: breast cancer**											
ERBB2	84	D	∙	✓	✓	FGFR1	50	D	∙	✓	✓
GAB2	35	∙	∙	∙	✓	PSG11	28	∙	∙	∙	✓
MACROD2	19	∙	✓	∙	✓	PTEN	16	D	✓	✓	✓
FRS2	10	∙	∙	∙	✓	IGF1R	10	∙	∙	∙	✓
CRK	10	∙	∙	∙	✓	JAK2	7	D	∙	✓	✓
AC116165.7-2	6	∙	✓	∙	∙	IRS4	6	∙	∙	✓	✓
PTK2	5	∙	∙	∙	✓	IL6ST	4	D	∙	✓	✓
EGFR	4	D	∙	✓	✓	GRB2	4	∙	∙	∙	✓
PTPN1	4	∙	∙	∙	✓	CREBBP	3	D/R	∙	✓	✓
DOK6	3	∙	∙	∙	✓	JAK1	2	D	∙	✓	✓
EGF	2	∙	∙	∙	✓	PIK3R1	2	R	∙	✓	✓
SYK	2	D	∙	✓	✓	PTPN6	2	∙	∙	✓	✓
VAV1	2	∙	∙	✓	✓						

Number of patients in the dataset that had a mutation in the gene is in c column. D/R stand for dominant or recessive otherwise unknown. Genes discovered by the quadratic mutual-exclusivity approach that does not include the network connectivity term are in QuanDMutEx column. COSMIC [26, 27] column represent if the gene present in COSMIC Cancer Gene Census. Genes present in DriverDBv2 [28] are in DDBv2 column

In the triple-negative breast cancer (TN) dataset, out of thirteen identified driver genes, eight are each mutated in only two out of 94 patients, and are analyzed below. A chromatin-remodeling gene CREBBP was found to be overexpressed in breast cancers [29], and is frequently mutated in bladder cancers [30]. DAPK1 is a potential tumor suppressor gene [31, 32]. NCOA1 was found to promote angiogenesis in breast cancers [33]. SLC39A7 is a potential oncogene in colorectal cancer [34]. IDH3B is upregulated in breast cancer and is significantly involved in energy metabolism in tumor progression [35, 36]. HIST1H4A is known to play a role in cell death induction in tumor cells [37]. HIF1A functions as a tumor promoter in cancer-associated fibroblasts, and as a tumor suppressor in breast cancer cells, also it is already a vaccine target in triple-negative breast cancer [38–40]. Finally, MLL methyltransferase are known to have a haematopoietic-specific tumorigenic capability [41].

In the ovarian cancer (HGS) dataset, twenty-three out of twenty-five identified genes are low-frequency driver genes – each is mutated only in two out of 316 patients. Of these twenty-three genes, CTNNB1 is implicated in malignant ovarian transformation [42]. DAG1 and HSPA5 are already drug targets [43, 44]. ERBB2, MST1R, STAT3, VAV3, ERBB3, NTRK2 and JAK2 are known oncogenes [45–50], and FANCA is a potential oncogene [51]. GRB2 is a therapeutic target for solid tumor prevention [52]. PIK3R1 represents a critical driver of endometrial cancer pathogenesis and is a therapeutic target [53]. TSHR signaling promotes the proliferation of ovarian cancer [54]. HSP90AA1 is considered as a potential protein target in therapy of ovarian cancer. [55]. UBC is potential drug resistance-related gene in ovarian cancer [56]. Finally, WRN and CDKN2A are tumor suppressor genes [57, 58].

In the glioblastoma multiforme (GBM) dataset, out of six identified driver genes, one is mutated in five, and two in only two out of 120 patients. Of these, MAPK9 was found to be significantly upregulated in glioma stem cells [59]. MDM2 is a known oncogene [60], while RPL11 is a tumor suppressor gene that acts together with MDM2 in p53 activation pathway [61]. In the METABRIC breast cancer dataset, out of twenty five genes identified by QuaDMutNetEx, six are very rare – each mutated in only two out of 696 patients. Of these, JAK1 is known for its key role in breast cancer progression [62]. EGFR signaling pathway also has a crucial role in mammary cancers [63], and polymorphism in the EGFR ligand, EGF, was found to affect cancer progression [64]. PIK3R1 and VAV1 are known oncogenes [65, 66], SYK is a tumor suppressor gene [67], and PTPN6 has a tumor suppressor role [68]. Together, these results confirm that QuaDMutNetEx is highly effective in identifying cancer driver genes with low mutation frequency.

For comparison, we used two network-based methods, DriverNet and HotNet2. We also used a mutual exclusivity tool, Dendrix. We ran the three tools on the same four datasets: TN, GBM, HGS, and METABRIC. DriverNet was designed to utilize genomic, transcriptomic, and biological network information, HotNet2 utilizes genomic and biological network information, while Denrix used only the genomic information. In all three methods, as well as in QuaDMutNetEx, we used the default parameters. For each method, we analyzed coverage, excess coverage, conformity to the mutual exclusivity pattern as quantified by the Dendrix score $n - \sum _{i=1}^{n} |G_{i} x - 1|$, and the number of connected components in the subgraph of the biological network consisting of the genes in the solution returned by the method.

### Testing in cancer molecular subtypes dataset

Mutual exclusive pattern in tumor can be resulted from other factors [69]. Hence, methods using mutual exclusivity need to account for that. Here we are using GBM subtypes to test the effectiveness of our method [69]. Using copy number, gene expression and methylation, GBM classified into proneural, neural, classical, and mesenchymal [70, 71]. We downloaded GBM data from TCGA and divided them into four subtypes according to TCGA IDs given in [71].

The genes discovered by QuaDMutNetEx are presented in Table 4. To evaluate the gene’s driver status, we used COSMIC Cancer Gene Census database [26, 27] and the cancer driver gene database DriverDBv2 [28]. All of the resulted genes exist in both COSMIC and DriverDB2 or one of them.

**Table 4:** Putative driver gene sets and metrics in GBM subtypes discovered by QuaDMutNetEx

Gene	c	D/R	COSMIC	DDBv2	Gene	c	D/R	COSMIC	DDBv2
**Classical GBM**									
**Metrics:**		samples	genes	mutations	Genes in solution	Coverage	Excess coverage	Connected components	
		n=69	p=487	1192	6	0.6232	0.1163	5	
EGFR	21	D	✓	✓	PCDHAC2	15	∙	∙	✓
DNAH9	4	∙	∙	✓	GABRA6	4	∙	∙	✓
PTPRG	2	∙	∙	✓	TEK	2	∙	∙	✓
**Mesenchymal GBM**									
**Metrics:**		samples	genes	mutations	Genes in solution	Coverage	Excess coverage	Connected components	
		n=75	p=510	1310	12	0.7733	0.1552	4	
PTEN	23	D	✓	✓	EGFR	17	D	✓	✓
PIK3CA	5	D	✓	✓	CPNE8	3	∙	∙	✓
KDM2B	3	∙	✓	✓	NRXN1	3	∙	∙	✓
INPPL1	3	∙	∙	✓	EZR	2	D	✓	✓
GRB10	2	∙	∙	✓	IRS1	2	D	✓	✓
IRS4	2	∙	∙	✓	LZTR1	2	D	✓	✓
**Proneural GBM**									
**Metrics:**		samples	genes	mutations	Genes in solution	Coverage	Excess coverage	Connected components	
		n=44	p=229	558	7	0.6364	0.0714	2	
TP53	15	R	✓	✓	PCDHAC2	5	∙	∙	✓
CHEK1	2	∙	∙	✓	CREBBP	2	D/R	✓	✓
DAXX	2	R	✓	✓	MECOM	2	R	✓	✓
TBP	2	∙	∙	✓					
**Neural GBM**									
**Metrics:**		samples	genes	mutations	Genes in solution	Coverage	Excess coverage	Connected components	
		n=41	p=199	482	8	0.6585	0.0370	2	
TP53	15	R	✓	✓	ANK2	5	∙	∙	✓
PDGFRA	2	D	✓	✓	FLT1	2	∙	∙	✓
PTPN11	2	D	✓	✓	CHD8	2	∙	∙	✓
DYNC1I1	2	∙	∙	✓	KDR	2	D	✓	✓

Boxes below the GBM subtypes show the metrics of a GBM subtype including number of samples, number of genes etc. Discovered genes by QuaDMutNetEx are below the metrics box. Number of patients in the dataset that had a mutation in the gene is in c column. D/R stand for dominant or recessive otherwise unknown. COSMIC [26, 27] column represent if the gene present in COSMIC Cancer Gene Census. Genes present in DriverDBv2 [28] are in DDBv2 column

### Comparison with existing methods

The quality of the solutions returned by QuaDMutNetEx is higher than solutions from other methods (see Table 5). QuaDMutNetEx consistently produces more mutually exclusive gene sets than the network-based methods, and the gene sets are more highly connected – the interaction networks have fewer separate connected components. Compared to Dendrix, the tool that only considers mutual exclusivity, QuaDMutNetEx solutions show slightly lower mutual exclusivity, but at the same time are much more highly connected.

**Table 5:** Comparison between QuaDMutNetEx, HotNet2, DriverNet, and Dendrix

Method	Genes in solution	Coverage	Excess coverage	Dendrix score	Connected components
TN: Triple negative breast cancer					
HotNet2	128	0.6809	0.7969	-118	9
DriverNet	21	0.6383	0.4667	23	14
Dendrix	22	0.6170	0.1034	51	8
QuaDMutNetEx	13	0.6854	0.0983	**55**	**3**
GBM: Glioblastoma multiforme					
HotNet2	37	0.7833	0.4149	10	11
DriverNet	17	0.9333	0.8661	-140	9
Dendrix	22	0.7166	0.023256	**84**	4
QuaDMutNetEx	6	0.8151	0.1855	79	**1**
HGS: high-grade serous ovarian cancer					
HotNet2	58	0.8449	0.4307	83	4
DriverNet	72	0.9335	0.6373	-35	51
Dendrix	3	0.8037	0.0	**254**	3
QuaDMutNetEx	25	0.6170	0.1086	236	**1**
METABRIC: breast cancer					
HotNet2	224	0.4424	0.7394	-1694	18
DriverNet	90	0.4683	0.7785	-1130	33
Dendrix	18	0.3836	0.1236	**233**	16
QuaDMutNetEx	25	0.3982	0.1753	216	**4**

### Effects of parameters on QuaDMutNetEx

The behavior of proposed method can be adjusted by three parameters, based on the knowledge of the tumor under study. Parameter *α* quantifies the reward for gene connectivity in cellular networks – higher values indicate stronger preference for finding densely connected genes. Parameter *k* controls how steeply the penalty for multiple mutations in a single pathway grows – lower values of *k* lead to lower penalization of excess coverage in relation to coverage, and is appropriate for slower growing tumors, where additional mutations in any given pathway have more time to accumulate by chance. Finally, higher values of parameter *C* penalize for solutions sets with many genes.

**Fig. 2:**
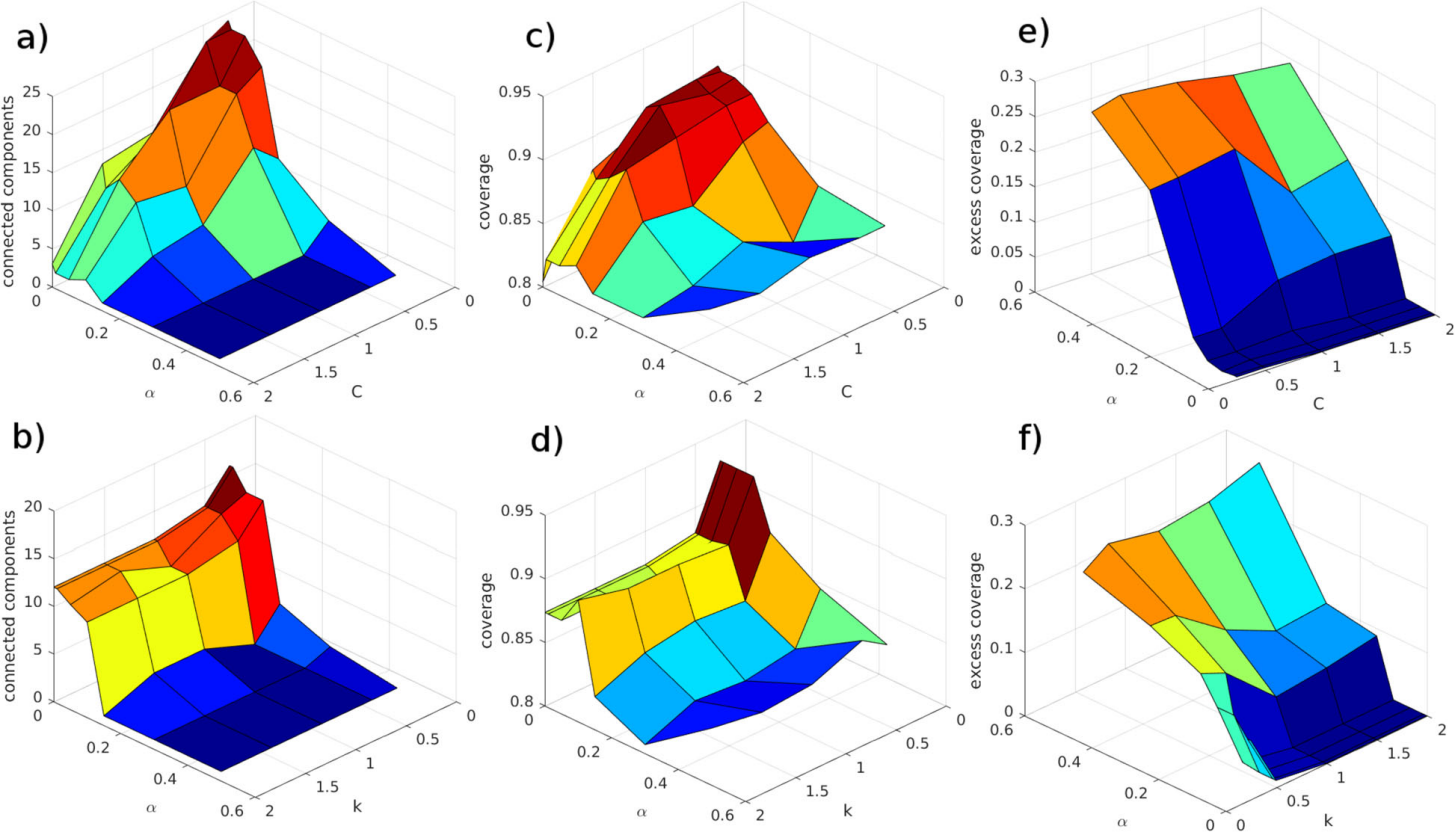
Effects of parameters on QuaDMutNetEx. **a**, **b**: effect on connected components; **c**, **d**: effect on coverage; **e**, **f**: effect on excess coverage. Results shown are for the HGS dataset, the results for other datasets are similar

We have analyzed how these parameters affect the solution by running QuaDMutNetEx for 100,000 iterations for parameters *α*=0.01,0.05,0.1,0.3,0.6,1 with *C*=0.25,0.5,1,1.5,2,2.5 and *α*=0.01,0.05,0.1,0.3,0.6,1 with *k*=0.25,0.5,1,1.5,2,2.5. Figure 2 shows that the parameter *α* achieves its design goal, that is, solution with higher *α* include fewer connected components and prefer connected network. The *α* parameter has the following effect on coverage and excess coverage: as the value of *α* increases, the coverage decreases and the excess coverage increases. Furthermore, as the value of *α* increases, it decreases the effect of *C* and *k*. Setting *α* to a low value, such as 0.001, makes the effect of *C* and *k* to be more dominant. Higher coverage and higher excess coverage, as expected, are observed for low *k* values. High values of *C* lead to solution sets involving only a few genes, while low values of *C* lead to high coverage.

## Discussion

The proposed method, QuaDMutNetEx, relies on two sources of information to detect cancer driver genes. It uses observed somatic mutations in a cohort of cancer patients, to detect mutual exclusivity patterns, and a biological network encoding functional relationships between genes to provide context for the observed data. Relying on two sources of information is a strength of the proposed method, since treated individually, each source is imperfect. Biological networks are know to be incomplete and contain false positives, and the functional, regulatory, and signaling relationships they capture are not all directly relevant to cancer. Mutual exclusivity patterns may not be perfectly present in the observed patient mutation data. This may be true especially for slower growing tumors, where the time from onset of the disease to its detection is long enough to allow for accumulation of additional mutations in functionally-related sets of genes that contribute to cancer. Depending on the knowledge of the analyzed type of cancer and characteristics of the studied patient cohort, the users of QuaDMutNetEx should adjust the parameters of the methods that govern the strength of preference for mutual exclusivity in relation to patient coverage, the weight assigned to the network knowledge, and the strength of preference for small driver gene sets. Users of QuaDMutNetEx should also keep in mind that it uncovers driver genes relevant to the cohort from which the mutation data comes from. That is, it detects drivers present in the particular set of patients, based on the particular type of mutation data provided.

## Conclusions

Experiments on four datasets show that QuaDMutNetEx has the ability to detect driver genes mutated with low frequency, genes that may be missed by existing tools that rely on mutual exclusivity alone, or on frequency and network information alone. Improvements in the quality and interpretability of the discovered putative driver gene sets makes QuaDMutNetEx a valuable addition to the family of driver discovery tools.

## Methods

### Input for QuaDMutNetEx

QuaDMutNetEx input has two sources of information. The first source is the binary somatic mutation matrix as in many mutual-exclusivity tools [15, 21]. Specifically, the data for *n* patients, each with total of *p* genes explored for possible existence of somatic mutations, arrives in a form of a mutation matrix *G*, an *n* by *p* sparse binary matrix. We expect *G*_*ij*_=1 if patient *i* has a somatic mutation in gene *j*, that is, a difference between cancer tissue and matched healthy tissue from the same patient is detected; otherwise, *G*_*ij*_=0. The change can be a point mutation in the coding region of the gene, potentially affecting its function. It could also be a mutation in the non-coding, regulatory elements of the DNA associated with the gene, or copy number alternation of the gene in case of homozygous deletions and high-level amplifications, affecting its expression. A row of the matrix describing mutations in patient *i* will be referred to as a vector *G*_*i*_. The second source of information is a gene connectivity matrix *A*, with nonzero *A*_*ij*_ values for genes *i* and *j* that are known to be related in a biologically meaningful way, for example one gene regulates the other, or proteins encoded by the genes are known to interact in a signaling pathway. The output of the method is a column vector *x* of length *p*, with *x*_*j*_=1 indicating that gene *j* is a putative cancer driver gene, that is, its mutations can contribute to cancer growth, and zero otherwise. The non-zero elements of the solution will be referred to as the solution gene set.

### Defining the quality of potential driver gene sets

For the binary solution vector *x* with length *p* genes, there are 2^*p*^−1 possible non-zero solution vectors, each encoding a different gene set. The challenge is to find a gene set that is composed of driver genes. To this end, we designed a penalty score that reflects how likely it is that genes encoded by a solution vector form a comprehensive set of driver genes impacting a single cellular function. The lower the penalty score, the more likely the solution consists of related driver genes. The penalty score has two major terms: a network term, and a mutual-exclusivity term.

**Fig. 3:**
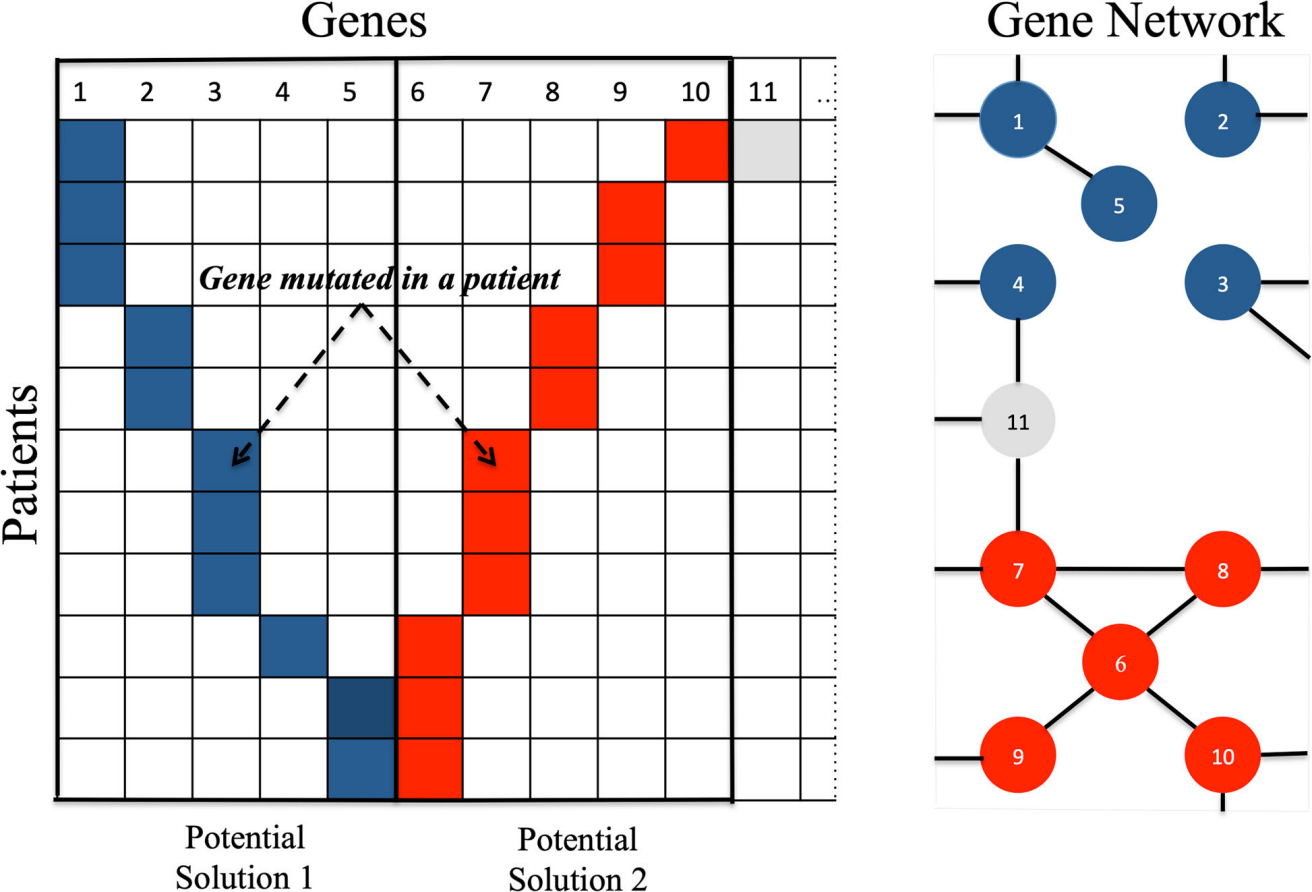
Illustration of the role of the network term *N*(*A*,*x*). Based solely on the mutual exclusivity, potential solutions 1 and 2 are equally good, both show perfect mutual exclusivity. Inclusion of network term *N*(*A*,*x*) makes potential solution 2 the preferred one, since it consists of more highly connected genes

The network terms captures our preference for solution gene sets in which the products of transcribing the genes are connected in known human protein-protein interaction networks. Other sources of pairwise gene relationships could be used, for example functional similarity, or regulatory interactions. This network connectivity preference is captured by a term *N*(*A*,*x*) in the objective function, where *A* is the undirected adjacency matrix corresponding to the network, and *x* is the solution gene set. The new term introduces a reward for any two genes in a solution that are connected. Since the solution vector is binary, the additional term can be defined as $N(A,x)=-x^{T}Ax=-\sum _{i,j} A_{ij} x_{i} x_{j}$. The scaled term *αN*(*A*,*x*) with nonnegative weight *α* corresponds to providing a reward of *α* every time two genes *i* and *j* present in the solution, that is, with *x*_*i*_=1 and *x*_*j*_=1, are connected by an edge, that is, when *A*_*ij*_=1. The effect of introducing the network term can be seen in Fig. 3.

The second term in the objective function captures mutual exclusivity pattern among solution genes. We use a flexible, quadratic term previously used in our mutual exclusivity method, QuaDMutEx [21]. Briefly, the term penalizes for solutions that leave some patients not showing any mutation in the solution genes, as well as for solutions in which patients are covered by more than one mutation. The penalty for excess mutations grows quadratically with the number of mutations over one. The ration of penalty for multiple mutations to penalty for no mutation can be tweaked by parameter *k*. For example, for a slow growing tumor, where there is ample time for additional mutations to accumulate in a single pathway, *k* should be low. In addition, we define parameter *C* to be a penalty incurred by adding one more gene to the solution set.

For any possible solution vector *x*, the penalty score is a sum of the two terms described above, and is

1 $$ {\begin{aligned} L(G,A,x)&=-\alpha x^{T} A x + \sum_{i=1}^{n} \frac{1+k}{2}\left(G_{i} x - 1\right)\left(G_{i} x - \frac{2}{1+k}\right) + C ||x||_{0}. \end{aligned}}  $$

### Algorithm for finding high-quality driver gene sets

The minimization of the quadratic penalty function *L*(*G*,*A*,*x*) over binary vectors *x* is an example of an unconstrained binary quadratic problem (BQP). While BQPs are known to be NP-hard [72], for problems small enough the optimal solution can still be found. For example, for datasets with up to 1000 patients, if one focuses on only *ν*=50 genes, the solution *x* that is the global minimum of *L*(*G*,*A*,*x*) can be found in below a second.

As we have shown before [21], high-quality approximate solutions to BQP problems involving thousands of genes can be found efficiently by an iterative algorithm that maintains an evolving set of size *ν* consisting of candidate driver genes, and in each of the *T* iterations finds an optimal solution to a small instance of the problem in Eq. 1 involving only the current candidate genes. This allows for improving the quality of the driver gene set, while exploring a diverse set of possible genes as candidates.

A single run of QuaDMutNetEx will return a set of functionally-related driver genes with high mutual exclusivity and high network connectivity. Running QuaDMutNetEx in sequence, removing discovered genes from input matrices *G* and *A* after each iteration, will allow to uncover genes from multiple pathways needed for oncogenesis, although the joint solution is no longer expected to have high connectivity, nor to conform to the mutual exclusivity pattern.

## Abbreviations

TCGA: The Cancer Genome Atlas
TN: Triple negative breast cancer
GBM: Glioblastoma multiforme
HGS: High-grade serous ovarian cancer
COSMIC: Catalogue of somatic mutations in cancer
DDBv2: DriverDBv2
